# Supplementary material for: The interactome of intact mitochondria by cross-linking mass spectrometry provides evidence for coexisting respiratory supercomplexes
Source: Mol Cell Proteomics. 2017 Dec 8;17(2):216–32. doi: 10.1074/mcp.RA117.000470 (PMC5795388; doi:10.1074/mcp.RA117.000470)
Supplement: Supplemental Data [file supp_RA117.000470_133922_0_supp_23978_fzffwf.zip › spectra_annotation/mito_DR_spectra_annotation/84-1-9-1-8-1.pdf]

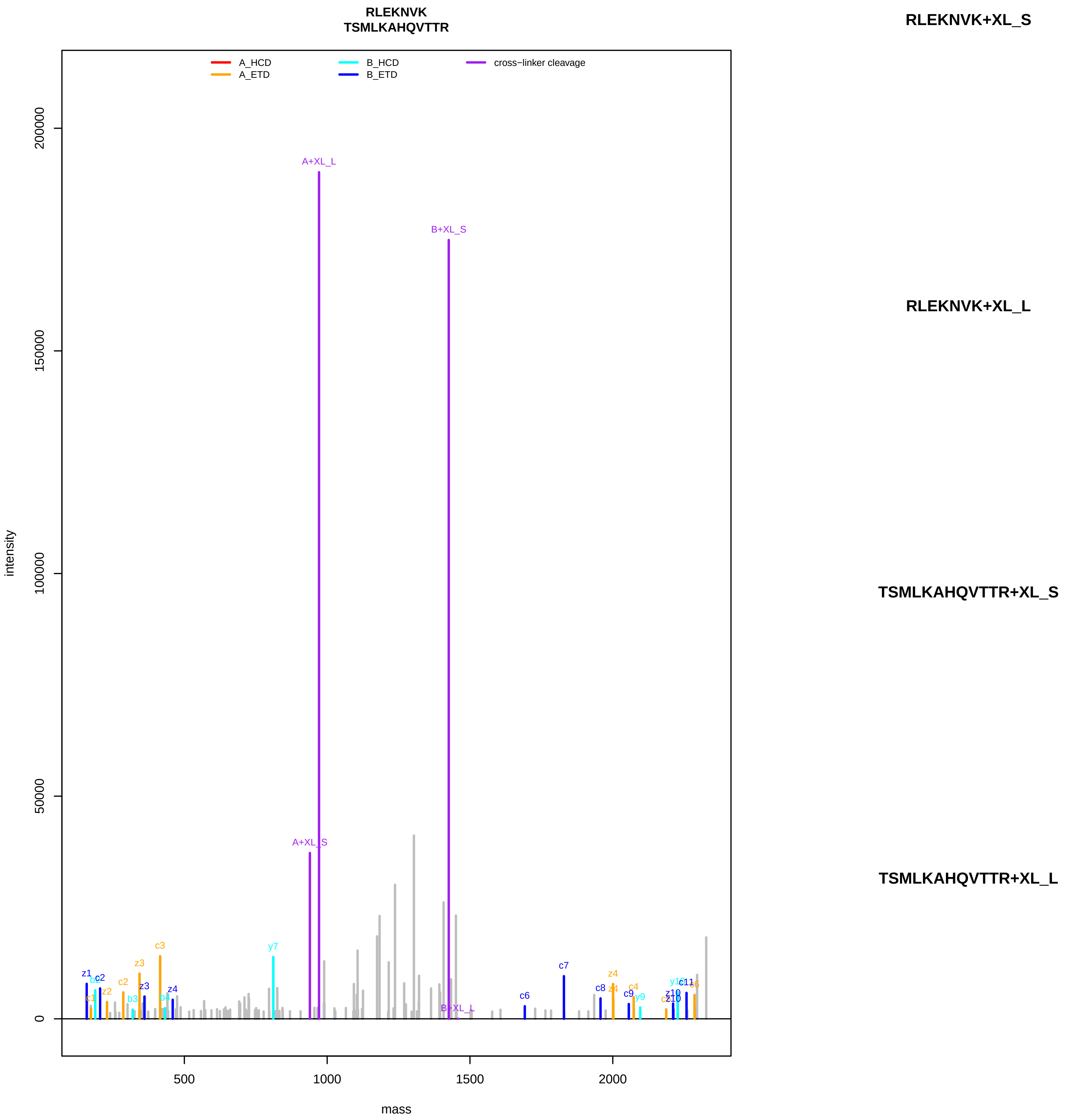
RLEKNVK
TSMLKAHQVTTR
A_HCD
A_ETD
B_HCD
B_ETD
cross−linker cleavage
A+XL_L
B+XL_S
A+XL_S
B+XL_L
intensity
mass
0
50000
100000
150000
200000
500
1000
1500
2000
z1
c2
z2
c2
z3
c3
z3
b3
z4
y7
c6
c7
c8
z4
c9
c4
y9
z10
c11
RLEKNVK+XL_S
RLEKNVK+XL_L
TSMLKAHQVTTR+XL_S
TSMLKAHQVTTR+XL_L
